# Fault Detection and Identification Method for Quadcopter Based on Airframe Vibration Signals

**DOI:** 10.3390/s21020581

**Published:** 2021-01-15

**Authors:** Xiaomin Zhang, Zhiyao Zhao, Zhaoyang Wang, Xiaoyi Wang

**Affiliations:** 1 School of Artificial Intelligence, Beijing Technology and Business University, Beijing 100048, China; zhangxiaomin@st.btbu.edu.cn (X.Z.); wangzhaoyang@btbu.edu.cn (Z.W.); wangxy@btbu.edu.cn (X.W.); 2 China Light Industry Key Laboratory of Industrial Internet and Big Data, Beijing Technology and Business University, Beijing 100048, China

**Keywords:** quadcopter, fault detection and identification, wavelet packet decomposition, LSTM network, airframe vibration signals

## Abstract

Quadcopters are widely used in a variety of military and civilian mission scenarios. Real-time online detection of the abnormal state of the quadcopter is vital to the safety of aircraft. Existing data-driven fault detection methods generally usually require numerous sensors to collect data. However, quadcopter airframe space is limited. A large number of sensors cannot be loaded, meaning that it is difficult to use additional sensors to capture fault signals for quadcopters. In this paper, without additional sensors, a Fault Detection and Identification (FDI) method for quadcopter blades based on airframe vibration signals is proposed using the airborne acceleration sensor. This method integrates multi-axis data information and effectively detects and identifies quadcopter blade faults through Long and Short-Term Memory (LSTM) network models. Through flight experiments, the quadcopter triaxial accelerometer data are collected for airframe vibration signals at first. Then, the wavelet packet decomposition method is employed to extract data features, and the standard deviations of the wavelet packet coefficients are employed to form the feature vector. Finally, the LSTM-based FDI model is constructed for quadcopter blade FDI. The results show that the method can effectively detect and identify quadcopter blade faults with a better FDI performance and a higher model accuracy compared with the Back Propagation (BP) neural network-based FDI model.

## 1. Introduction

The quadcopter, with its vertical take-off and landing capability, simple mechanical structure, and easy maintenance, has been widely used in a variety of military and civilian mission scenarios, such as search and rescue [1,2], package delivery [3], border patrol [4], military surveillance [5], and agricultural applications [6,7]. Abnormal and unexpected situations, such as actuator failure, sensor failure, and structural failure could occur during the flight of the quadcopter. The real-time online Fault Detection and Identification (FDI) of the abnormal state of the quadcopter is vital for the safe flight of aircraft. The current available methods for Unmanned Aerial Vehicle (UAV) fault diagnosis can be basically divided into three categories: analytical model-based methods, knowledge-based methods, and signal processing-based methods [8,9,10,11,12].

The analytical model-based method focuses on using an accurate mathematical model and observable inputs and outputs to construct residual signals. By analyzing the residual signals, the difference between the expected behavior of the system and the actual operation mode realizes the FDI of the UAV. Such methods mainly include state estimation methods [13,14,15,16,17], parameter estimation methods [18,19,20,21,22,23,24], etc. The FDI methods based on state estimation are mainly based on filters and observers, which realize the state estimation of the system and determine whether it is within the allowable thresholds. Mehra et al. [25] firstly employed Kalman filtering in fault diagnosis to estimate the state of the system. Liu et al. [26] designed multiple fault detection filters for each component of the multiple fault signals that may occur in the system. By evaluating the residuals of fault thresholds, the proposed design can effectively detect multiple faults in a quadcopter system. Chen et al. [27] established a mathematical model of quadcopter UAV and proposed an adaptive observer-based quadcopter fault diagnosis method. The simulation results verify the effectiveness of the designed robust nonlinear controller and fault estimation scheme. Avram et al. [28] presented an FDI method for quadcopter based on nonlinear adaptive estimation which systematically designed adaptive thresholds to achieve enhanced robustness and fault sensitivity at fixed thresholds. Zhong et al. [29] presented a method for quadcopter fault detection and diagnosis based on an adaptive three-stage Kalman filter which could significantly reduce the computational effort and effectively distinguish between aircraft vehicle faults and external interference. FDI methods based on parameter estimation are performed by detecting parameter changes in the model. Yoon et al. [30] presented a hybrid FDI scheme for three consecutive faults of UAV tilt inertial sensors. The combination of the odd-even space method and the in-lane monitoring method improved the system tolerance to multiple consecutive failures during flight. The performance of the proposed FDI scheme was validated by hardware-in-the-loop tests and fixed-wing UAV flight tests. Since it is difficult to establish accurate mathematical models of objects in some practical applications, the analytical model-based method has limitations to its implementation in many cases.

Knowledge-based FDI methods mainly use the knowledge of systems established by experts in this field. The simulation of expert reasoning processes can realize system FDI. Such methods include FDI methods based on expert systems [31], fuzzy inference [32], and fault trees [33]. A fuzzy fault tree analysis algorithm was proposed in the literature [34]. From the perspective of providing the failure possibility of the underlying event, the knowledge and experience of experts are integrated to calculate the failure interval of the system components. By directly calculating the fuzzy fault tree interval, fuzzy reliability interval, and traditional reliability, the faulty system can be effectively diagnosed. In terms of the complexity, diversity, and nonlinearity of UAV system faults, Xiao et al. [35] proposed an FDI method based on a combination of expert system and BP network which overcomes the lack of effective self-learning in traditional expert systems. The diagnosis example of UAV telemetry and remote control system shows that the expert system can effectively diagnose the UAV system and has a good application prospect in the field of FDI. Due to the complexity of knowledge-based methods, the FDI accuracy highly depends on the level of expert knowledge. Meanwhile, the FDI speed decreases with the rule number. Thus, the efficiency of the knowledge-based FDI method needs to be improved.

Signal processing-based methods extract the features of the measurement signals to achieve FDI without establishing complex mathematical models of the system. Due to the widespread existence of vibration signals in rotating machinery, it is widely used in the FDI of rotating machines. Such methods include the wavelet transform method [36], the spectral analysis method [37], etc. Glowacz et al. [38] proposed a device to analyze the vibration signal of a three-phase induction motor. The vibration signal and signal processing methods are used to diagnose the rotor of the three-phase induction motor, and the diagnosis effect is good. Caesarendra et al. [39] proposed a parsimonious network based on a fuzzy inference system and tested the data of low-speed slewing bearings. This method is applied to the normal to faulty bearing vibration data collected in 139 days to predict the time series feature of the vibration signal of slewing bearing. The outstanding performance of the network is verified through experiments. Guo et al. [40] proposed an FDI method for UAV sensors based on hybrid feature models and deep learning. The method firstly collected the remaining signals of different sensor failures, such as global positioning system, inertial measurement unit, air data system, etc. Then, it transformed the residual signal into a corresponding time-frequency diagram by short-time Fourier transform. Through a convolutional neural network, it finally extracted map features for the FDI of the UAV sensors.

Due to the limited space of the quadcopter airframe, it is difficult to use additional sensors to capture fault signals. Therefore, we adopt limited airborne sensor data to achieve FDI without building a complex mathematical model of a quadcopter. In this paper, a signal processing-based method is introduced to detect and identify quadcopter blade faults. Yan et al. [41] established a quadcopter FDI model based on the BP network using the vibration signals of quadcopters. However, the model only adopts single-axis vibration data, which makes it difficult to identify different faults on different axes of quadcopters. In addition, the BP network is affected by the local minimization problem as the weight of the network is gradually adjusted in the direction of local improvement. Local minimization means that the weight converges to a local minimum and network training fails. A quadcopter FDI model based on a BP network has limited data mining capabilities, which makes it sensitive to initial network weights. Initializing the network with different weights tends to converge to different local minima, resulting in different training results each time and reducing the accuracy of the model. When the amount of vibration data is relatively large and the failure modes are relatively large, the BP-based FDI model cannot effectively mine the data information and has limitations in practical applications.

Considering the deficiencies, this paper proposes a quadcopter FDI method based on airframe vibration signals using airborne acceleration sensors without additional sensors. First, the three-axis accelerometer data of the quadcopter from the flight experiment is regarded as the airframe vibration signal. Second, the wavelet packet decomposition method is employed to extract the data features and the standard deviations of the wavelet packet coefficients are employed to compose the feature vector. Finally, an FDI model is established by LSTM network that realizes the detection and identification of the propeller blade faults of a quadcopter.

## 2. FDI Algorithm

In this section, we introduce the FDI algorithm, including the feature extraction of vibration signals based on wavelet packet decomposition, and the FDI model establishment of a quadcopter based on an LSTM network.

### 2.1. Algorithm Overview

**Figure 1:**
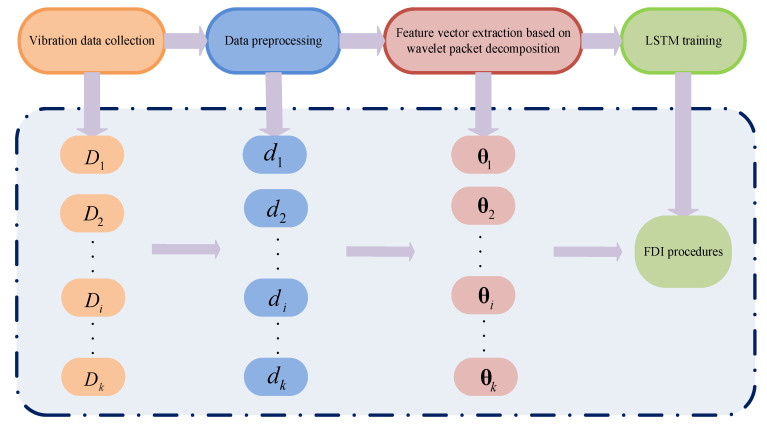
Flow chart of the algorithm.

The quadcopter FDI method based on the vibration signals of the airframe is shown in Figure 1. The method includes vibration data acquisition, data preprocessing, feature extraction, and LSTM-based FDI model training. First, the accelerometer sensor obtains airframe vibration data of the quadcopter during flight. The collected data sets are divided into *K* sets according to *K* health states during the flight of the quadcopter, which forms data sets $D_{i}(i=1,2,\cdots ,K)$, respectively. Each data set is divided into multiple subsets by dividing subsets into units of 1 s, and *K* data sets are preprocessed to obtain data sets $d_{i}(i=1,2,\cdots ,K)$. Then, the wavelet packet decomposition extracts the features of the data set $d_{i}(i=1,2,\cdots ,K)$. The matrix composed by a feature vector is written as $\theta_{i}(i=1,2,\cdots ,K)$, which is imported to train the LSTM network model that realizes the quadcopter FDI based on airframe vibration signal.

### 2.2. Signal Feature Extraction

#### 2.2.1. Decomposition of the Airframe Vibration Signal

**Figure 2:**
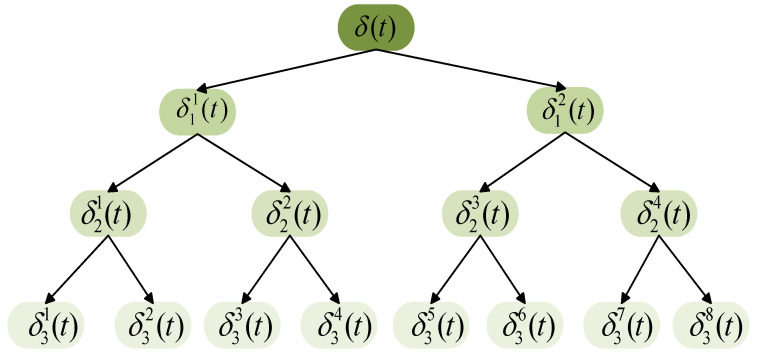
Wavelet packet dendrogram.

Signal processing can iteratively optimize the existing signal and eliminate the redundant part of the signal. Fourier transform analyzes the signal by mapping the time domain to the frequency domain with a large span; however, the volatility of the signal cannot be reflected in the vibration signal of the quadcopter airframe [42]. Because wavelet packet decomposition can describe whether the signal has fluctuations and characterize the locality of the signal, it can extract the characteristics that are weakened or even ignored and research in many fields involve the relevant content of wavelet packet transform [43,44,45]. The wavelet packet decomposition suits signal processing, especially non-stationary signals. It can provide a higher resolution in the high-frequency region and better analyze the signal according to its characteristics. We select a condensed part that can describe all the signals to replace the whole with a part. The condensed part refers to the data features extracted by the wavelet packet decomposition. The extracted features and constructed feature vectors are employed to describe the whole dataset. The three-level wavelet decomposition method in wavelet packet decomposition can meet the detection requirements. Taking the three-level wavelet decomposition as an example, the original signal is decomposed into eight wavelet component signals. Figure 2 depicts the three-level wavelet packet decomposition tree, where $\delta_{j}^{m}(t),j=1,2,3;m=1,2,\cdots ,8$ denotes the *m*-th wavelet packet component signal of the *j*-th level.

If the *j*-level wavelet packet decomposition method is applied to the original airframe vibration signal δ(t), the original airframe vibration signal δ(t) is:

(1) $$\delta (t)=\sum_{m=1}^{2^{j}}\delta_{j}^{m}(t).$$

A wavelet packet is a function with three indices of integers, *m, j*, and *l*, which are the modulation, scale, and translation parameters, respectively [46]. The wavelet packet component signal $\delta_{j}^{m}(t)$ can be expressed by the linear combination of the wavelet packet function $\psi_{j,l}^{m}(t)$ as:

(2) $$\delta_{j}^{m}\left(t\right)=\sum_{l=-\infty }^{+\infty }p_{j,l}^{m}\left(t\right)\psi_{j,l}^{m}\left(t\right).$$

The wavelet packet coefficient $p_{j,l}^{m}(t)$ is obtained by:

(3) $$p_{j,l}^{m}\left(t\right)=\int_{-\infty }^{+\infty }\delta \left(t\right)\psi_{j,l}^{m}\left(t\right)dt.$$

Assuming that the wavelet packet function is orthogonal, then:

(4) $$\psi_{j,l}^{r}\left(t\right)\psi_{j,l}^{s}\left(t\right)=0,if\left(r\neq s\right).$$

The method focuses on the feature extraction of the *N*-axis airframe vibration signal of the quadcopter. Suppose that:

(5) $$\delta_{i}^{n,t}=[\delta (1),\delta (2),\cdots ,\delta (\lambda )]\in {\mathbb{R}}^{1\times \lambda }(i=1,\cdots ,K,n=1,\cdots ,N,t=1,\cdots ,q),$$

where *K* denotes the number of data sets and *N* denotes the number of axes. Divide the data set into q subsets by time, and λ is the number of data points in the *n*-th axis airframe vibration signal of the *i*-th data set at the *t*-th time period. $\delta_{i}^{n,t}$ denotes observations in the *n*-th axis airframe vibration signal of the *i*-th data set at the *t*-th time period.

Then, the *N*-axis airframe vibration signal of the *i*-th data set at the *t*-th time period is:

(6) $$\delta_{i}^{t}={\left[\delta_{i}^{1,t},\delta_{i}^{2,t},\cdots ,\delta_{i}^{N,t}\right]}^{T}\in {\mathbb{R}}^{N\times \lambda }.$$

Finally, the *N*-axis airframe vibration signal of the quadcopter in the data set $d_{i}$ is:

(7) $$\delta_{i}=[\delta_{i}^{1},\delta_{i}^{2},\cdots ,\delta_{i}^{q}]\in {\mathbb{R}}^{N\times \lambda \times q}.$$

#### 2.2.2. Feature Extraction of the Airframe Vibration Signal

A common method for the extraction of the characteristics of the original airframe vibration signal regards the energy of the wavelet packet coefficients as the feature vector, but training artificial neural network by the standard deviation of wavelet packet coefficients has a faster convergence speed and better performance [46]. Therefore, we adopt the standard deviation of wavelet packet coefficients to construct the feature vector to train the LSTM-based FDI model.

The preprocessed quadcopter *N*-axis airframe vibration signal in (7) adopts the *j*-level wavelet packet decomposition method. Decompose the *n*-th axis airframe vibration signal of the *i*-th data set at the *t*-th time period $\delta_{i}^{n,t}$ in the data set $d_{i}$. The wavelet packet coefficient $p_{j,l}^{m}(t),m=1,2,\cdots ,2^{j}$ can be obtained by Equations (1)‒(3). Then, the standard deviation of wavelet coefficient constructs the *M*-dimensional feature vector:

(8) $$\theta_{i}^{n,t}={\left[\sigma_{1},\sigma_{2},\cdots ,\sigma_{M}\right]}^{T}\in {\mathbb{R}}^{M\times 1},$$

where:

(9) $$\sigma_{m}=\sqrt{\frac{1}{M}\sum_{k=1}^{M}{\left(p_{j,l}^{m}-\mu \right)}^{2}},M=2^{j},m=1,2,\cdots ,M.$$

μ denotes the mean of the wavelet packet coefficient $p_{j,l}^{m}$ and $\theta_{i}^{n,t}$ denotes the *M*-dimensional feature vector constructed by the *j*-level wavelet packet decomposition method in the *n*-th axis vibration signal of the *i*-th data set at the *t*-th time period.

Then, we have:

(10) $$\theta_{i}^{t}={\left[\theta_{i}^{1,t},\theta_{i}^{2,t},\cdots ,\theta_{i}^{N,t}\right]}^{T}\in {\mathbb{R}}^{N\times M},$$

where $\theta_{i}^{t}$ denotes the feature matrix extracted by the *N*-axis vibration signal of the *i*-th data set at the *t*-th time period.

The *N*-axis vibration signal of the quadcopter in preprocessed data set $d_{i}$ is subjected to feature extraction, and we obtain the input matrix of the LSTM-based FDI model as:

(11) $$\theta_{i}=[\theta_{i}^{1},\theta_{i}^{2},\cdots ,\theta_{i}^{q}]\in {\mathbb{R}}^{N\times M\times q}.$$

### 2.3. LSTM-Based FDI Model of Quadcopter

**Figure 3:**
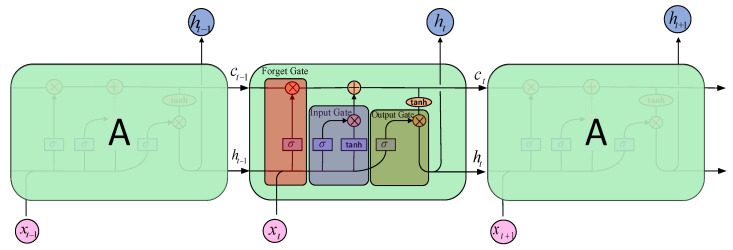
LSTM structure diagram.

Hochreiter et al. [47] proposed LSTM, a special recurrent neural network, which can solve vanishing gradient. Schematic diagram of a LSTM unit is shown in Figure 3.

LSTM maintains the hidden state vector $h_{t}$ and a memory cell $c_{t}$. The behavior of the memory cell is determined by three gates—i.e., the input gate $i_{t}$, the output gate $o_{t}$, and the forget gate $f_{t}$. The updated equations are given as follows:

(12) $$f_{t}=\sigma (V_{f}h_{t-1}+W_{f}x_{t}+b_{f}),$$

(13) $$i_{t}=\sigma (V_{i}h_{t-1}+W_{i}x_{t}+b_{i}),$$

(14) $$o_{t}=\sigma (V_{O}h_{t-1}+W_{o}x_{t}+b_{o}),$$

(15) $$\tilde{c_{t}}=\tanh(V_{c}h_{t-1}+W_{c}x_{t}+b_{c}),$$

(16) $$c_{t}=f_{t}⊙c_{t-1}+i_{t}⊙\tilde{c_{t}},$$

(17) $$h_{t}=o_{t}⊙\tanh(c_{t}),$$

where $x_{t}$ denotes the new input to the LSTM model at time step *t*; $V_{\Theta },W_{\Theta },b_{\Theta }$ are learnable parameters (Θ can be f,i,o, or c); and σ is nonlinear function (σ here usually it is sigmoid function). The operator ⊙ denotes the element-wise multiplication. At time step t in (12), a function of the new input and previous hidden state $h_{t-1}$ forms the forget gate $f_{t}$, determining which historical information will be discarded by the cell state. In (13), the input gate $i_{t}$ is obtained through a function of the new input and the previous hidden state $h_{t-1}$, deciding which states will be updated. In (14), a function of the new input and the previous hidden state $h_{t-1}$ forms the output gate $o_{t}$. Then, (15) calculates the candidate values that will be added to the new cell state described by (16), together with the values of the old cell state which are regulated by the forget gate. Finally, the output gate decides which part of the new cell states should be taken to form the new hidden state $h_{t}$, as shown in (17).

**Figure 4:**
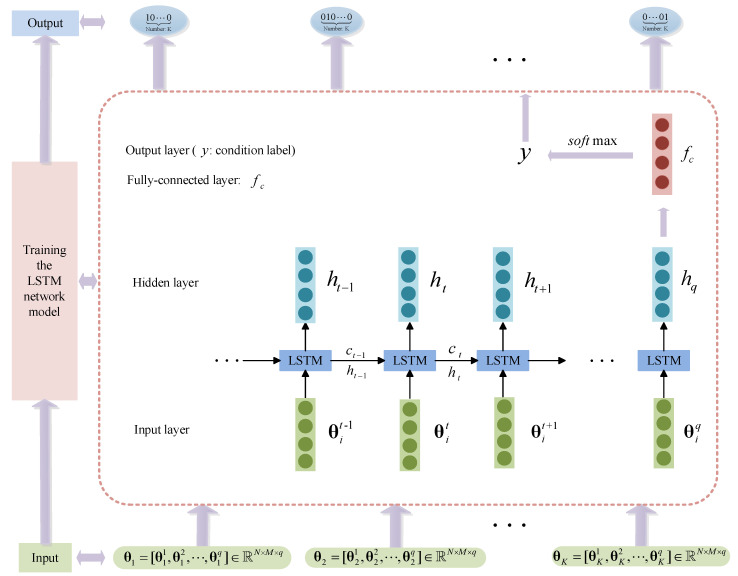
LSTM-based FDI model.

Feature extraction is performed in the *N*-axis vibration signal of the quadcopter for the preprocessed data set $d_{i}(i=1,2,\cdots ,K)$. When the standard deviation of the wavelet coefficients constructs the *M*-dimensional feature vector, we finally obtain the input matrix $\theta_{i}$ of the LSTM-based FDI model, as shown in (11). The matrix $\theta_{i}^{t}$ is conducted as the input unit of the input layer to train the LSTM-based FDI model. We employ *K* health states of quadcopter blades as the output of the LSTM-based FDI model. By training the LSTM-based FDI model, the failure degree of the blades of the quadcopter is identified. Figure 4 shows the LSTM-based FDI model in this paper.

This model incorporates wavelet packet decomposition and LSTM and applies it to the research on quadcopter blade FDI. According to the characteristics of the data-driven model, the dynamic system feature extraction of wavelet packet decomposition and the dynamic system anomaly detection of LSTM are proposed. The FDI method effectively solves the FDI problem of the blades for the quadcopter.

## 3. Experimental Validation

In this section, the vibration data of three axes X, Y, and Z directions (*N* = 3) of the quadcopter blades under three health states (*K* = 3) are collected, with non-damaged blades, 5% broken blades, and 15% broken blades, where 5% and 15% refer to the damaged part of the blade, accounting for 5% and 15% of the blade mass, respectively. The three-level wavelet packet decomposition method (*M* = 8) further processes the data and extracts data features, which train and further verify the LSTM-based FDI model. The experiment results are compared with the quadcopter FDI method based on the BP network.

### 3.1. Collection and Preprocessing of Airframe Vibration Data

In order to meet the data requirements for training neural networks, it is necessary to measure the flight vibration data of the quadcopter in different states. As a platform, we use the Parrot AR.Drone, a commercially available quadcopter, which measures 53 cm × 52 cm and weights 420 g. Its main advantage is its very low price, its robustness to crashes, and the fact that it can safely be used indoors and close to people. The AR.Drone is equipped with a 3-axis gyroscope and accelerometer, an ultrasound altimeter, and two cameras. The first camera is aimed forward, covers a field of view of 73.5° × 58.5°, and has a resolution of 320 × 240. The video of the first camera is streamed to a laptop at 18 fps, using lossy compression. The second camera aims downward, covers a field of view of 47.5° × 36.5° and has a resolution of 176 × 144 at 60 fps. The onboard software uses the down-looking camera to estimate the velocity. The quadcopter sends gyroscope measurements and the estimated velocity at 200 Hz to the laptop.

**Figure 5:**
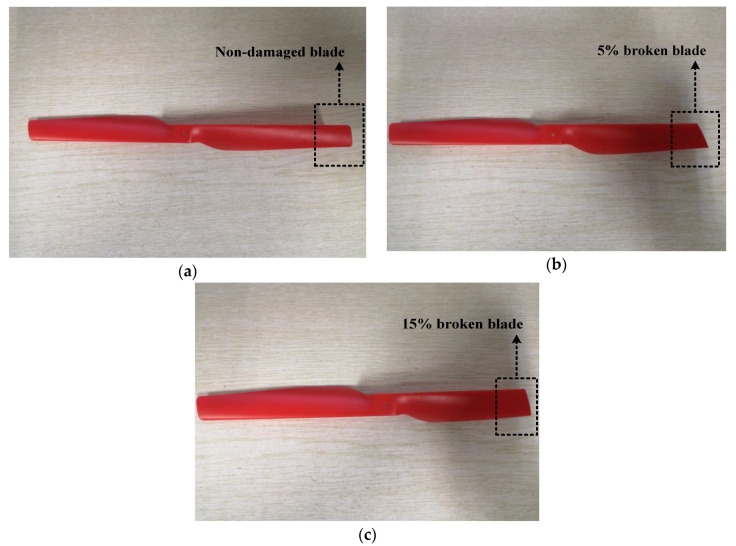
Quadcopter blades under three health states. (**a**) Non-damaged blade, (**b**) 5% broken blade, (**c**) 15% broken blade.

**Figure 6:**
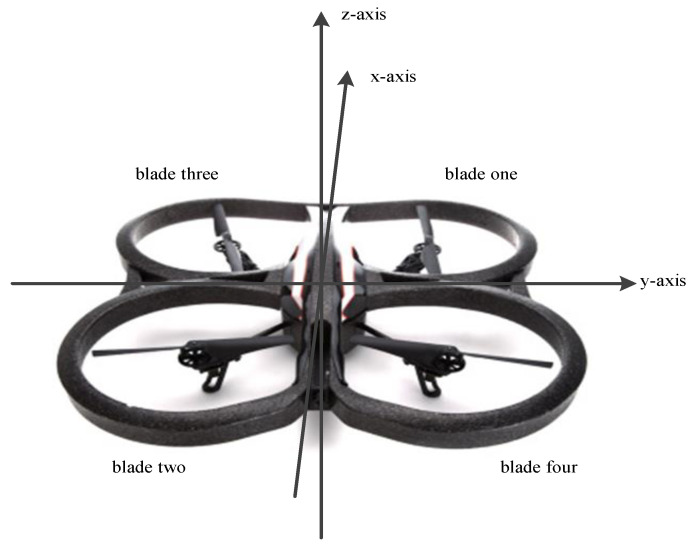
The structure diagram of the quadcopter and the x, y, and z-axis regulations.

**Figure 7:**
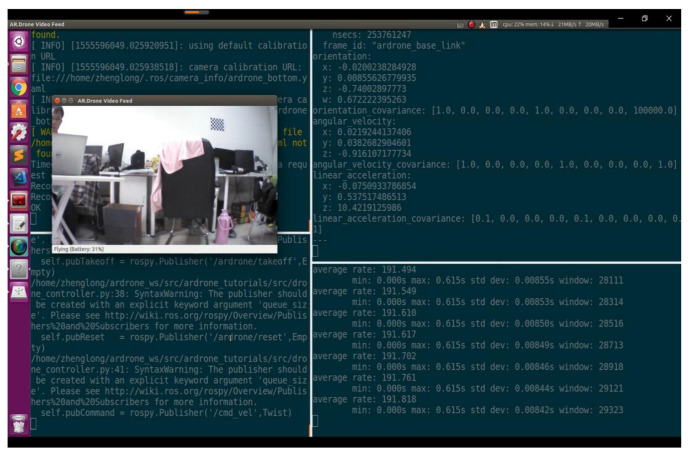
The monitoring screen of real-time communication between the wireless communication module on the AR.Drone platform and the laptop.

The three health states of the quadcopter blades in this experiment are non-damaged blades, 5% broken on blade one and blade two, and 15% broken on blade two and blade four, as shown in Figure 5. The structure diagram of the quadcopter and the x, y, and z-axis regulations are shown in Figure 6. According to the positive direction of the x-axis, starting from the rightmost and counterclockwise, they are marked as blade one, three, two, four. Perform flight experiments separately. The wireless communication module on the AR.Drone platform communicates with the laptop in real time. We obtained the images transmitted back by the quadcopter during flight and the vibration data of the quadcopter. The monitoring screen is shown in Figure 7.

The flight vibration data were collected at a sampling frequency of 200 Hz/s. The number of original data collected were 95,508 pieces of non-damaged blades data, 28,284 pieces of 5% broken blades data, 25,475 pieces of 15% broken blades data. The measured flight vibration data under three health states are collected in data set D1, data set D2, and data set D3, respectively. The blades of a quadcopter provide lift, which directly affects the acceleration and attitude angle. When the blade fracture of the quadcopter is not serious, and the aircraft is flying smoothly, the acceleration fluctuation is not large. Angular velocity is an important component of the quadcopter’s freedom, which can completely depict the flight state of the entire quadcopter body. Therefore, we only need to extract the angular velocity of x, y, and z-axis from the flight vibration data of quadcopter. Data preprocessing includes two steps. The first step is to remove invalid data and acceleration data in data set D1, data set D2, and data set D3 caused by the data acquisition software. The second step is to divide data set D1, data set D2, and data set D3 into 477, 141, and 127 subsets, respectively, taking one second as a unit and 200 pieces of data as a group. Finally, the preprocessed data set is rewritten into d1, d2, and d3, and the data samples of d1 are shown in Table 1.

### 3.2. Signal Feature Extraction and LSTM-Based FDI Model Design

Three-level wavelet packet decomposition is performed on the angular velocity data of the x, y, and z axes in each subset of d1, d2, and d3. The angular velocity data of each axis of each subset are decomposed into eight wavelet component signals, and 8-dimensional feature vectors are constructed by the standard deviation of the wavelet packet coefficients. Therefore, the x, y, and z axes generate a total of 24-dimensional feature vectors. Extract 477 feature vectors from d1, of which 277 construct matrices $\alpha_{1}$ and 200 construct matrices $\alpha_{2}$. Extract 141 feature vectors from d2, of which 91 construct matrices $\beta_{1}$ and 50 construct matrices $\beta_{2}$. Extract 127 feature vectors from d3, of which 77 construct matrices $\gamma_{1}$ and 50 construct matrices $\gamma_{2}$. $\alpha_{1},\beta_{1},\gamma_{1}$ are training samples. $\alpha_{2},\beta_{2},\gamma_{2}$ are test samples. The training samples are ${\left[{\left[\alpha_{1}\right]}_{3\times 8\times 277},{\left[\beta_{1}\right]}_{3\times 8\times 91},{\left[\gamma_{1}\right]}_{3\times 8\times 77}\right]}_{3\times 8\times 445}$ and test samples are ${\left[{\left[\alpha_{2}\right]}_{3\times 8\times 200},{\left[\beta_{2}\right]}_{3\times 8\times 50},{\left[\gamma_{2}\right]}_{3\times 8\times 50}\right]}_{3\times 8\times 300}$.

Specifically, for three-axis data of the quadcopter x, y, and z, respectively, the input matrix of non-damaged blades is 8×477, the input matrix of 5% broken blades is 8×141, and the input matrix of 15% broken blades is 8×127. The output is divided into three types with the output definitions shown in Table 2, and the training design is shown in Table 3, where M represents the dimension and q represents the number of samples.

### 3.3. Training and Verification of LSTM-Based FDI Model

In order to verify whether the LSTM-based FDI model can solve the time series classification problem and identify faulty quadcopter blades, this experiment selects different features under the same LSTM-based FDI model as an input. We select the feature input method with the highest correct rate and verify the correctness of the training algorithm in this paper.

**Figure 8:**
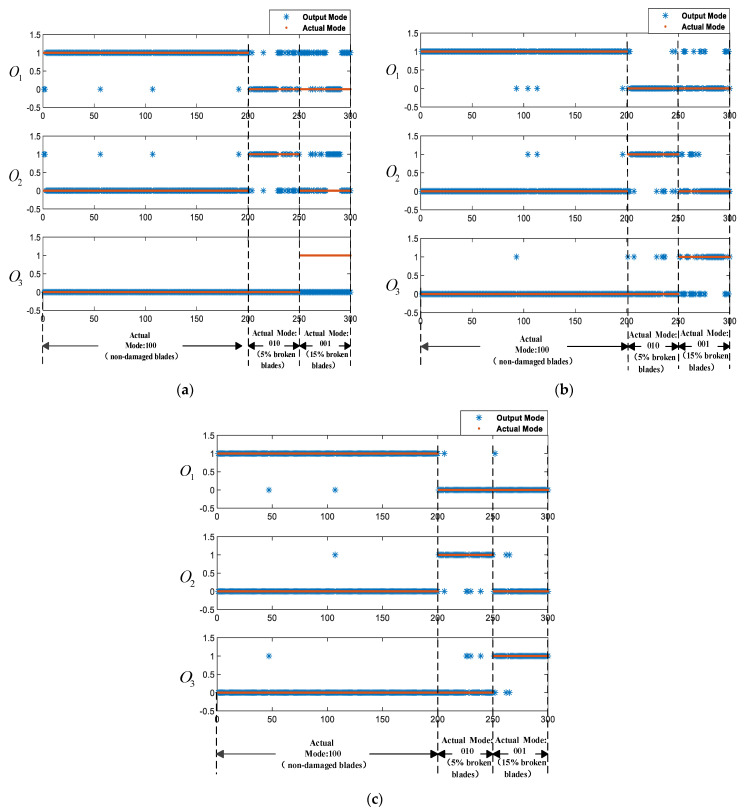
The output results of the three methods: (**a**) FDI results of method 1, (**b**) FDI results of method 2, (**c**) FDI results of method 3.

From freedom degree of the quadcopter, multiple combinations and different combinations would cause various results when selecting data training of different axes. This experiment discusses three fusion methods—i.e., the single x-axis angular velocity feature vector is the input of network in method 1; the x and y axes angular velocity feature vector is the input of the network in method 2; and the x, y, and z axes angular velocity feature vector is the input of the network in method 3. The training methods and accuracy rates are shown in Table 4, where N represents the number of axis, M represents the dimension, and q represents the number of samples. The output results of three methods are shown in Figure 8a–c, where the X-axis represents the number of input test sample sets and the Y-axis represents the number of output codes.

The experiment results show that the accuracy rates of method 1, method 2, and method 3 are 75%, 91.33%, and 96%, respectively. As shown in Figure 8a, some of the FDI results of method 1 are wrong. When 5% are broken on blade one and blade two and 15% are broken on blade two and blade four, the vibration signal in the x-axis does not change significantly. Only the single x-axis angular velocity feature vector is used as the input to train the LSTM-based FDI model, which makes the training result reliability and the accuracy rate decrease, so method 1 is discarded. Although the combination of two-axis can detect the blade failure status of quadcopter in Figure 8b,c, the fusion of angular velocity data of three-axis is more accurate. When two or three-axis data are fused, the amount of data increases and the flight attitude of the aircraft is more complete. The data features that can be learned by the algorithm are richer. Additionally, the algorithm can mine more data characteristics in the same unit of time. In summary, the dynamic system abnormal state detection model based on LSTM is highly reliable. Method 3, the angular velocity data fusion scheme of the x, y, and z axes, is better.

### 3.4. Comparison of LSTM and BP Based FDI Models

The BP-based FDI model can also detect and identify the failure of the quadcopter. This experiment compares the LSTM-based FDI model with the BP-based FDI model in terms of time series classification. The reliability of the LSTM training algorithm in FDI has been obtained. We compare the advantages and disadvantages of the LSTM-based FDI model with the BP-based FDI model under the same input. The LSTM-based FDI model selects method 3. The training design of the BP-based FDI model is shown in Table 5.

**Figure 9:**
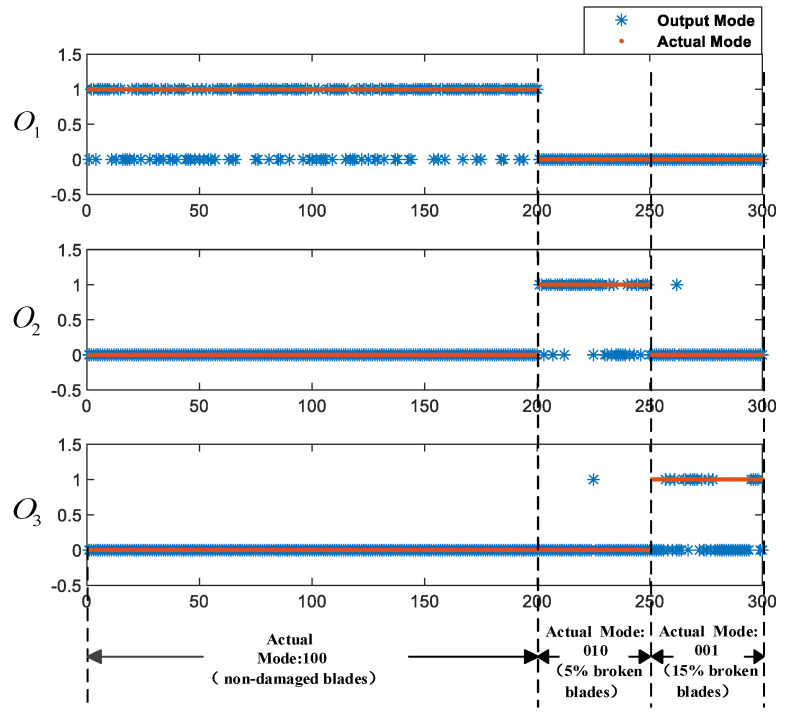
FDI results of the BP-based FDI model.

The experiment results of the LSTM-based FDI model are shown in Figure 8c. The experiment results of the BP-based FDI model are shown in Figure 9.

It can be seen from Figure 8c and Figure 9 that the experiment results of the LSTM-based FDI model are basically in line with expectations, and the accuracy rate is as high as 96%. However, the accuracy rate of the BP-based FDI model in identifying the blade failures of the quadcopter is only 65%.

Among the quadcopter FDI methods, the LSTM-based FDI model outperforms the BP-based FDI model in time series classification, with a higher accuracy and better generalization ability. It can quickly adjust the learning rate to find the upper and lower bounds of the dynamic learning rate.

## 4. Conclusions

Aiming at the FDI of the quadcopter, this paper proposes an FDI method based on airframe vibration signal. This method integrates multi-axis data information and effectively detects and identifies quadcopter blade faults through an LSTM-based FDI model. The quadcopter triaxial accelerometer data are collected for airframe vibration signals, the wavelet packet decomposition method is employed to extract data features, and the standard deviations of the wavelet packet coefficients are employed to form the feature vector. The FDI model is constructed based on LSTM and compared with the BP-based FDI model. The experiment results show that the proposed method has a higher accuracy in the FDI of quadcopters based on airframe vibration signals as the accuracy increases to 96%. When the amount of vibration data is relatively large, the BP-based FDI model cannot effectively mine the data information, and the accuracy of the model is only 65%. Therefore, it can only judge part of the degree of blade fracture data correctly, and the model accuracy is lower than that of the LSTM-based FDI model. Future work may include the blade FDI of other multi-rotor aircraft, such as hexacopters and octocopters. In addition, locating faults through vibration signals is the focus and difficulty of further research. Additionally, more fault modes, such as sensor fault, are expected to be incorporated into the established model. The proposed method in this paper can combine the iterative schemes [48,49,50,51,52,53] and recursive schemes [54,55,56,57,58,59,60,61,62] to study the parameter identification problems of linear and nonlinear stochastic systems with colored noises [63,64,65,66,67,68,69,70,71] and to present highly efficient fault detection methods that can also be applied to the literature.

## Figures and Tables

**Table 1:** Data samples of d1.

Time	Angular Velocity X	Angular Velocity Y	Angular Velocity Z
1,555,587,861.30445	0.158639818429947	0.0292043387889862	0.0286896377801895
1,555,587,861.31157	0.142599448561668	0.0653511583805084	0.0378986895084381
1,555,587,861.31336	0.175936982035637	0.0409883372485638	0.010272428393364
1,555,587,861.31747	0.16002993285656	0.0935227274894714	0.0228112936019897
1,555,587,861.32163	0.100399397313595	0.0828494876623154	0.0202383417636156
1,555,587,861.33311	0.139274418354034	0.0667824372649193	0.0288971811532974
1,555,587,861.33323	0.141123101115227	0.0438796132802963	0.0646019577980042
1,555,587,861.33652	0.272148668766022	0.0218221284449101	0.0085440427064896
1,555,587,861.34083	0.198535457253456	0.0541263408958912	0.0249678939580917
1,555,587,861.34948	0.193962648510933	0.0859876573085785	−0.007232904434204

**Table 2:** LSTM output definitions.

$O_{1}$	$O_{2}$	$O_{3}$	Indication
1	0	0	non-damaged blades
0	1	0	5% broken blades
0	0	1	15% broken blades

**Table 3:** Training design.

Training Group	Data Set d1	Data Set d2	Data Set d3
Training samples [M × q]	x axis: $[8\times 277]{\;}^{1}$	x axis:${\left[8\times 91\right]}^{\;2}$	x axis: $[8\times 77]{\;}^{3}$
	y axis:$[8\times 277]{\;}^{1}$	y axis:$[8\times 91]{\;}^{2}$	y axis:$[8\times 77]{\;}^{3}$
	z axis:$[8\times 277]{\;}^{1}$	z axis:$[8\times 91]{\;}^{2}$	z axis:$[8\times 77]{\;}^{3}$

^1^ represents the matrix of non-damaged blades, ^2^ represents the matrix of 5% broken blades, and ^3^ represents the matrix of 15% broken blades.

**Table 4:** Three methods and accuracy.

Categories	Method 1 (x axis)	Method 2 (x, y axis)	Method 3 (x, y, z axis)
Training samples [N × M × q]	$[8\times 277]{\;}^{1}$	${\left[16\times 277\right]}^{\;1}$	$[24\times 277]{\;}^{1}$
	$[8\times 91]{\;}^{2}$	$[16\times 91]{\;}^{2}$	$[24\times 91]{\;}^{2}$
	$[8\times 77]{\;}^{3}$	${\left[16\times 77\right]}^{\;3}$	$[24\times 77]{\;}^{3}$
Test samples [N × M × q]	$[8\times 200]{\;}^{1}$	$[16\times 200]{\;}^{1}$	$[24\times 200]{\;}^{1}$
	$[8\times 50]{\;}^{2}$	$[16\times 50]{\;}^{2}$	${\left[24\times 50\right]}^{\;2}$
	$[8\times 50]{\;}^{3}$	$[16\times 50]{\;}^{3}$	$[24\times 50]{\;}^{3}$
Accuracy	75%	91.33%	96%

^1^ represents the matrix of non-damaged blades, ^2^ represents the matrix of 5% broken blades, and ^3^ represents the matrix of 15% broken blades.

**Table 5:** The training design of the BP-based FDI model.

Training Group	Data Set d1	Data Set d2	Data Set d3
Training samples [M × N × q]	$[24\times 277]{\;}^{1}$	$[24\times 91]{\;}^{2}$	${\left[24\times 77\right]}^{\;3}$
Test sample [M × N × q]	$[24\times 200]{\;}^{1}$	$[24\times 50]{\;}^{2}$	$[24\times 50]{\;}^{3}$
Output result ($O_{1}O_{2}O_{3}$)	100	010	001

^1^ represents the matrix of non-damaged blades, ^2^ represents the matrix of 5% broken blades, and ^3^ represents the matrix of 15% broken blades.

## Data Availability

The data presented in this study are available on request from the corresponding author. The data are not publicly available due to privacy.
